# Effects of a parental support intervention for parents in prison on child-parent relationship and criminal attitude—The For Our Children’s Sake pragmatic controlled study

**DOI:** 10.1371/journal.pone.0283177

**Published:** 2023-03-23

**Authors:** Åsa Norman, Pia Enebrink

**Affiliations:** Department of Clinical Neurosciences, Karolinska Institute, Stockholm, Sweden; VIVE - The Danish Center for Social Science Research, DENMARK

## Abstract

**Background:**

Children of incarcerated parents run a high risk for poor health and marginalisation across development where positive parenting comprises an essential protective factor. The For Our Children’s Sake (FOCS) intervention is delivered with incarcerated parents in Sweden to support parenting and healthy child development. This study aimed to explore the effects of the FOCS intervention on relationship quality between parent and child, parent criminal attitude and interest in treatment, while investigating intervention fidelity.

**Methods:**

The non-randomised non-blinded pragmatic controlled study was carried out during 2019–2020 in 15 prisons with 91 parents throughout Sweden. Group allocation was based on the set operation planning at each prison. Prisons delivering FOCS during the study period were recruited to the intervention group, whereas prisons delivering FOCS later were recruited to the control group. Outcomes were measured through parent-report at baseline September-December 2019 (T0), after intervention (T1) in January-April 2020, and at three-months follow-up in April-July in 2020 (T2). The primary outcome was relationship quality between incarcerated parent and child and secondary outcomes were criminal attitude, interest in other treatment programmes, and child-parent contact. Fidelity to intervention delivery was monitored through objectively rated audio recorded sessions by researchers, and by group-leader-reported logs. Group differences on outcome over time and at each time point were explored using mixed-model regression with repeated measures with an intention-to-treat approach and per protocol.

**Results:**

The intention-to-treat analysis showed favourable intervention effects over time for relationship quality, explained by a higher intervention group score at T2. An intervention effect was found for parental interest in other prison-delivered treatments at T2. The analysis per protocol found similar but stronger effects on the relationship quality and an additional intervention effect over time for criminal attitude, also explained by a significant group difference at T2. The effect on treatment interest did not reach statistical significance in the analysis per protocol. Group leaders reported that all sessions had been performed and the objective ratings of fidelity rendered overall acceptable delivery of the intervention.

**Conclusions:**

The FOCS intervention had beneficial effects on relationship quality, and outcomes related to criminality which suggests that a parenting intervention for incarcerated parents has the potential to influence both parenting outcomes and outcomes related to a criminal lifestyle. Future studies should investigate intervention effectiveness on long-term outcomes related to both child health and parental recidivism. Further development of intervention components is suggested with the hypothesis to increase intervention effectiveness.

**Trial registration:**

ClinicalTrials.gov: No. NCT04101799, prospectively registered on September 24, 2019, Identifier: https://clinicaltrials.gov/ct2/show/NCT04101799, The authors confirm that all ongoing and related trials for this intervention are registered.

## 1. Introduction

Children of incarcerated parents comprise a disadvantaged group with high risk of marginalisation and poor well-being [1–3], and incarceration also inflicts negative emotions in relation to parenting for the incarcerated parent [4, 5]. In an international perspective, children with incarcerated parents have a higher risk of a number of negative outcomes related to health and marginalisation, such as mental, social, emotional, behavioural, and physical problems compared to children without incarcerated parents [1–3, 6–8]. In addition, cross-country data including Swedish data, point towards an intergenerational effect on delinquency carried from parent to child, where children of incarcerated parents run a higher risk for own delinquency compared to children without incarcerated parents [2, 9, 10]. Studies conducted in Sweden also show that these children have a higher risk for antisocial behaviour, poor academic achievement, poor well-being and mental health, delinquency, teenage pregnancy, and unemployment across development, compared to children without an incarcerated parent [2, 10–12]. Qualitative studies from Sweden reveal that that these children experience the separation from the parents, as a result of incarceration, as traumatic. Also, the children describe how they, on the one hand, try hard to keep the parent’s incarceration a secret, and on the other hand, how they are stigmatised and bullied because of the parent’s incarceration when it has been revealed to others. The children also voice a need for support in understanding the situation as well as for emotional support [12]. Furthermore, in the perspective of the incarcerated parent, the forced separation from the child during incarceration can inflict despair, powerlessness, and stress related to parenting [4, 5].

Positive parenting is an essential component for a healthy and thriving progression in the development of a child [13]. However, incarcerated parents may be restricted in their capability to perform positive parenting, or in the understanding of what positive parenting is, due to a number of reasons. These reasons may coincide with risk factors for delinquency such as drug addiction, poverty, or negative family factors such as lack of experience of positive parenting in their own childhood [14]. Interventions that focus on family factors such as positive parenting have been suggested to support the promotion of healthy child development for children of incarcerated parents and to prevent the intergenerational effect of criminality [9, 15].

Scientific evaluations of interventions to support parenting and child development specifically developed for incarcerated parents suggest improvements in outcomes related to parenting [16–18], but also to child behaviour [16, 17, 19], and have also indicated a decrease in parental recidivism [20]. These programmes often include elements that focus on practicing positive parenting behaviour, such as altering or learning a new behaviour through parenting skills training (e.g., positive and encouraging communication with the child, healthy family routines, and effective problem solving) [16, 21]. Although a number of interventions for incarcerated parents have been developed and evaluated on the international arena, no such scientific evaluation has been undertaken in neither the Swedish nor the Northern European context to date. In 2021, 0.5 ‰ of the population in Sweden were incarcerated (n = 5689), with 94% men and 6% women, and about 60% with a sentence of four years or less [22]. It is uncommon for children to live with their parents in prison, although it is allowed for children <1 year of age. In Sweden, similar to other Northern European countries, the prison and probation context has a solid focus on supporting prisoners towards building their own capacity to leave criminality and enter a life as non-criminal citizens in society after the imprisonment. Thus, the focus on rehabilitation within Swedish prisons includes engaging prisoners in activities that promote behaviour change, education, or professional skills and focus on the rehabilitation of prisoners. As this focus may differ from the prison and probation contexts in other countries, internationally developed interventions may be difficult to generalise to the Swedish context, and therefore the For Our Children’s Sake (FOCS) parenting programme was developed specifically for parents incarcerated in Swedish prisons with the aim to support positive parenting for the child’s healthy development. FOCS was developed in 2012–2014. Since 2015, FOCS has been used in Swedish prisons and funded by tax money from the Swedish state, as all prison activities in Sweden. FOCS was subsequently implemented in prisons in Sweden but without scientific evaluation of possible, either positive or harmful, intervention effects. It is of great importance to explore the effects of an intervention which is to be used as standard practice in order to determine that the intervention has the intended beneficial effects, and that it does not harm participants. Therefore, the aim of this project was to evaluate the effects of the FOCS parenting programme on relationship quality between parent and child, parent criminal attitude and interest in treatment, and child-parent contact frequency through a controlled study while monitoring fidelity to intervention delivery. We hypothesised that the intervention group would have a significantly higher score on the primary outcome child parent relationship quality, and on the secondary outcomes interest in participating in other treatment programmes and frequency of parent-child contact, and a lower score on criminal attitude compared to the control group after the intervention.

## 2. Methods

### 2.1. Study design

The effectiveness evaluation of the FOCS intervention was carried out as a non-randomised and non-blinded pragmatic controlled study during 2019–2020. It was not possible to randomise group allocation due to the structure of how the Swedish Prison and Probation Service (SPPS), responsible for all Swedish prisons, is organised. The SPPS is a state authority for which yearly directives, including funding and core activities, are given by the Swedish government. Based on the government’s directive, the SPPS further provides instructions and funding to each prison yearly. The prison subsequently prepares an operation and economic planning for the year ahead, which includes all activities that are to be conducted in the specific prison during the coming year. At the time of this study, the plannings of the prisons were already finalized and thus stated whether a prison would carry out FOCS or not. Therefore, group allocation was based on the set operation planning at each prison. Thus, the prisons that agreed to participate in the study and where FOCS was carried out during the study period, in accordance with the operation planning, were included in the intervention group, whereas prisons that agreed to participate in the study and that planned for FOCS later on were included in the control group. No matching of groups was performed. A study protocol which described the evaluation of the intervention has been published and this reporting of the trial follows the TREND guidance [23] (S1 File). The trial was prospectively registered in ClinicalTrials.gov: No. NCT04101799, Identifier: https://clinicaltrials.gov/ct2/show/NCT04101799.

### 2.2. Setting, recruitment, and participants

The study setting comprised prisons in Sweden on high-, medium-, and low security levels for either men or women. In Sweden, prisons are run by the governmental authority SPPS, and thus all prisons are state prisons without exceptions. Prisons are specific for women and men, but inmates with a variety of sentences can be placed in the same prison. Normally, with the exception for bad behaviour, inmates are transferred to lower security prisons in a stepwise manner during their sentence, in line with the overall focus on rehabilitation within the SPPS. All inmates have compulsory, scheduled activity in prison for six hours per day during weekdays. Activities include occupational activity, studies on elementary to university level, and rehabilitation programmes. About 26% of inmates participate in rehabilitation programmes. These are manual based, commonly include cognitive behavioural therapy focusing on target behaviours such as criminality, violence, or drug abuse, and can be either in individual or group format [22]. All visits must first be granted by the specific prison. Most visits then take place in specific rooms where the inmate and visitor/s are allowed privacy.

**Fig 1:**
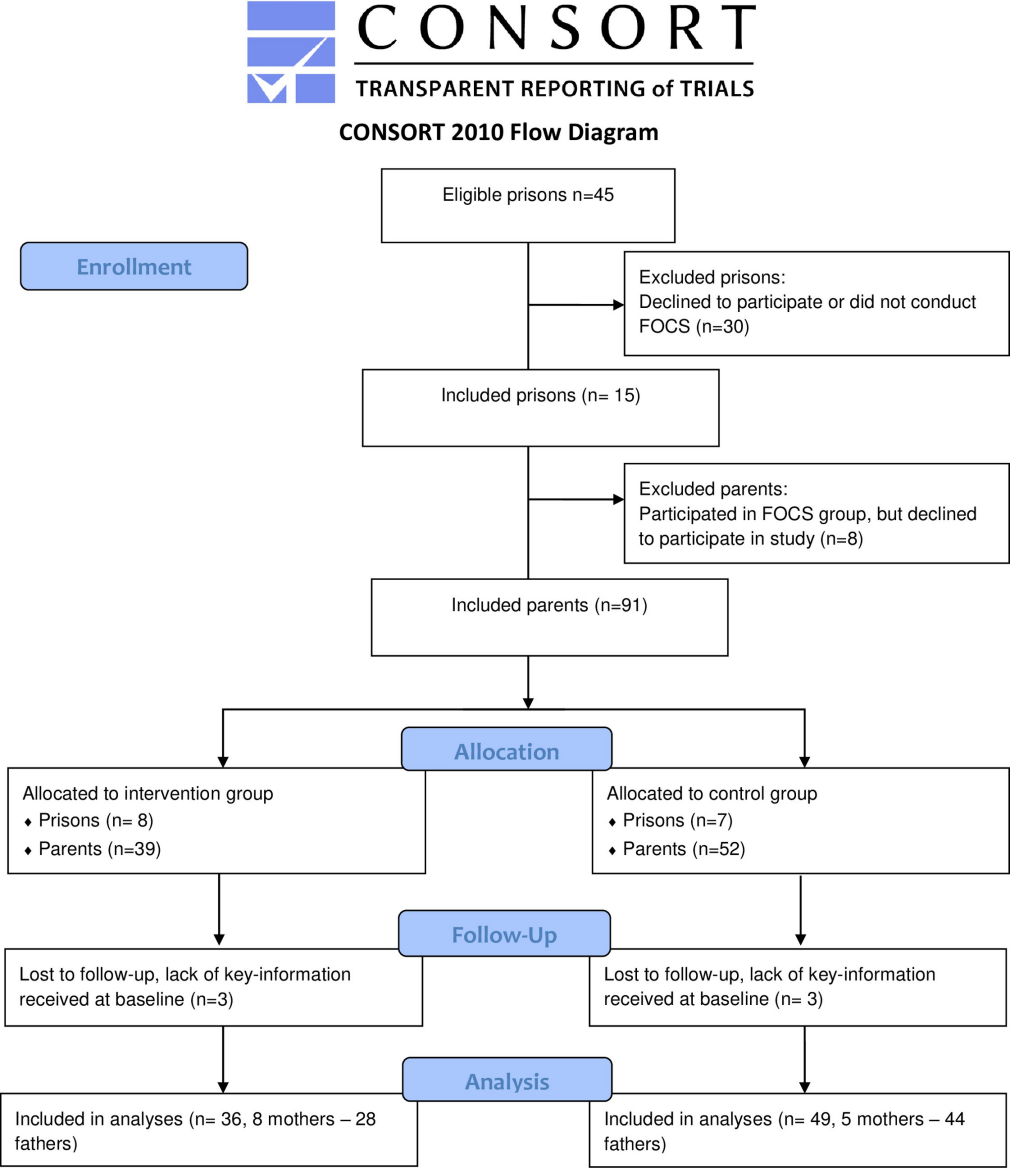
CONSORT 2010 flow diagram.

In this study, recruitment of prisons and participants was undertaken as follows. First, all prisons with trained group leaders or a plan to train group leaders during 2019–2020 were invited to participate. Prisons that agreed to participate (n = 15, intervention = 8, control = 7) were allocated to intervention or control condition based on the operation planning as described above (see Fig 1). Second, group leaders in the included prisons identified eligible parents among the prison inmates and informed the parents about the study and possibility to participate. Inclusion criteria for parents comprised having at least one child in the ages between 0–18 years, legal right to be in contact with the child, and not having committed a crime against the child or any violent crime against the other parent (to avoid parent-child contact that could be harmful for the child). In addition, the parent had to have sufficient proficiency in Swedish to be able to actively participate in the intervention. Parents who were not in contact with their child/ren or had children in the ages 0–2 or 13–18 years were only included in the secondary measurements as the primary measurement, quality in relationship between parent and child, assumes parent-child contact and is developed for children aged 3–12 years. In total, 91 parents were included in the study (see Fig 1). Prisons in the intervention group completed one FOCS group each, i.e., 8 FOCS groups in total. Written consent was obtained by all parents prior to participation and the consent has been provided in detail in the published study protocol [24]. Ethical approval was granted by the Swedish Ethical Review Authority 2019–04227.

### 2.3. Exposure—the “For Our Children’s Sake” intervention

The overall aim of the FOCS intervention is to support positive parenting for children’s healthy development. The intervention has been described in detail in the study protocol [24] and will be described briefly here. FOCS was developed for mothers and fathers in the Swedish prison context specifically as a collaborate project by the SPPS and the non-governmental organisation working with children of incarcerated parents, BUFFF, in 2012–2014. The FOCS programme is based on developmental psychology, attachment theory, social cognitive theory, and the Convention on the Rights of the Child [25] and includes manualised group leader material, group-leader training, and participant material. The intervention is delivered by two group leaders as weekly, two-hour group sessions for ten weeks where each session highlights a specific focus related to child development (e.g., children’s age specific needs, risk- and protective factors for healthy development), child perspectives on issues such as parental incarceration or violence (e.g., how children are affected by parental incarceration and/or violence and their reactions and needs, the parent’s own expression of anger), parenting (e.g., roles and responsibilities of a parent) and factors related to the own parenting (e.g., meaning, feelings and goals related to parenting and the children, cooperation and communication with the co-parent, reunion after incarceration). The material includes information regarding children of all ages (0–18 years) as FOCS groups commonly include participants with children of different ages. The content of the intervention has been described in more detail in the study protocol [24]. Group activities focus on group members’ reflections and joint discussion on the sessions’ themes and do not include skill related training such as role plays etc. Discussions are facilitated by open-ended questions and information in the group leader manual, or by theme related video films. As prisons are separated for men and women, the groups were delivered for mothers or fathers separately. Group leaders are commonly employed as prison staff but can also be external staff from children’s rights organisations. All group leaders have completed a five-day group leader training given by head trainers employed by the SPPS. The training covers the theoretical background of FOCS, contents of the material, and mode of delivery. Group leaders in this study were employed as either correctional officers or treatment staff (whose main duties are to conduct the rehabilitation programmes), of whom the majority were women and born in Sweden, but where there was a variation in educational background and age among group leaders.

The control condition received treatment as usual related to child-parent activities according to the available activities in each prison. These activities are few and infrequent for the most part, and the availability of activities differ between prisons. The activities include the parent recording bedtime stories for the child to listen to, spending visits with the child in a family furnished room in the prison, and parenting focused one-on-one counselling provided non-governmental organisations working for children’s rights, once per month at the most. Parents in the control condition were offered to participate in the FOCS programme after the follow-up measurement in accordance with the operation planning at each prison.

### 2.4. Data collection

#### 2.4.1. Outcome data

Primary and secondary outcome data were collected at baseline, before the intervention started at each prison, during September-December 2019 (T0), after intervention (T1) in January-April 2020, and at three-months follow-up in April-July in 2020 (T2).

*Primary outcome*. The primary outcome comprised relationship quality between incarcerated parent and child measured by the sub-scale closeness of the widely used instrumented Child Parent Relationship Scale (CPRS) [26]. The closeness sub-scale measures the degree of affection, warmth, and open communication between the parent and child with good internal consistency (alpha = 0.74) in a previous study which included parents of four-to six-year-olds [26]. In this study, internal consistency using Cronbach’s alpha was T0 = 0.81, T1 = 0.73, and T2 = 0.83. Parents in the study who had a child aged 3–12 years self-reported the measurement on a five-point Likert scale, and the mean score of the seven items was used for the study. If a parent had more than one child in the target ages, an index child for which the parents responded to the scale at all three time points, was chosen randomly by the research staff.

*Secondary outcomes*. Secondary outcomes were responded to by all parents in the study, regardless of the age of their child/ren. All secondary outcomes were self-reported and included parental criminal attitude, parental interest in other treatment programmes within the SPPS, and contact frequency between incarcerated parent and child/ren.

Criminal attitude was measured with the sub-scale antisocial intent of the Measures of Criminal Attitudes and Associates (MCAA) scale. The scale has previously been validated in a Swedish context, including a prison population, with good validity and reliability [27], and internal consistency using Cronbach’s alpha in this study was T0 = 0.84, T1 = 0.79 and T2 = 0.86. Parents responded on a five-point Likert scale and the mean score of the eight items was used in this study.

Interest in other treatment programmes within the SPPS was measured using one item specifically designed for this study:”How interested are you generally in participating in the SPPS treatment programmes (do not count FOCS)?”. Parents responded on an 11-point scale (0 no interest, 10 very high interest).

Contact frequency between parent and child was measured as intervals: no contact, seldom (less than once per month), at least once per month, or at least once per week. As the contact between child and parent is influenced by prison regulations and not always a result of the parent’s actions, this measurement was analysed in a general manner, where a dichotomised variable displaying contact at least once per week or less than once a week was used.

#### 2.4.2. Demographic measurements

Demographic data were acquired at T0 and encompassed parental characteristics including sex, age, region of birth, education, main source of income, and status of co-habitation with child and co-parent before incarceration, age, sex and number of children, custody of children, number of convictions, and length of current conviction.

#### 2.4.3. Process data

Data regarding the intervention process were collected during the intervention. Dose of received intervention was monitored as the number of sessions that the parents attended which was noted by the group leaders. Fidelity to the intervention delivery as performed by group leaders was monitored in two manners: group leader-report in a log and researcher-coding of recorded intervention sessions. The logs were developed by the researchers and filled in by the group leaders after each session. The log included information about whether central themes were covered during the session in accordance with the programme manual, the length of the sessions, and attendance of group leaders. In addition, all sessions were audio-recorded and a random selection of 31 out of the total of 45 recordings (70%) were coded for additional measures of fidelity of intervention delivery using the Fidelity of Implementation Rating System (FIMP) [28]. The FIMP rating system was developed for fidelity ratings of group leaders delivering parenting programmes according to the model Parent Management Training Oregon (PMTO) and has shown good predictive validity [29]. The FOCS programme has a different theoretical base and intervention structure than PMTO programmes, where the latter focuses on teaching parenting skills, providing feedback and instructions for parenting behaviour in interaction with the child. This type of skills training is not possible in the prison context as the child-parent contact is limited, and FOCS focuses on promoting discussion and reflection about positive parenting. Therefore, three of the original five dimensions of FIMP were deemed suitable for the rating of FOCS sessions: knowledge (of the programme and underlying theory), structure (balancing activities and keeping an orderly flow), and clinical process (creating a safe and supportive context). Ten-minute segments corresponding to the central themes in the sessions as stated in the log (see above) were identified, where the sessions included 3–5 segments depending on the number of central themes. Each segment was then coded for the three dimensions: knowledge, structure, and process on a 9-point scale from “needs work–insufficient adherence” with ratings of 1 to 3, “acceptable work–adequate competence/adherence with some mistakes” with ratings of 4 to 6, and “good work–quality competence/adherence rating” with ratings of 7 to 9. Three coders trained in using FIMP to identify segments in FOCS sessions and to rate segments for the three dimensions until inter-rater reliability with intra class correlation (ICC) of 0,7 was reached, with a total of approximately 40 hours of training. Two of the coders then rated the sessions over a period of four weeks. A third (30%) of the 31 sessions were coded by both raters and weekly meetings to monitor inter-rater reliability and discuss difficulties were held in which the third rater provided supervision. A total ICC between the two raters over the four-week rating period per dimension was: Knowledge—ICC 0.96, Structure—ICC 0.97, Process—ICC 0.97.

### 2.5 Statistical analysis

The software IBM SPSS, version 27.0 (Chicago, Illinois, USA), was used for all statistical analyses and analyses were carried out non-blinded. The level of significance was set to 0.05. Baseline differences between groups were tested using independent sample t-test and Mann-Whitney U-Test.

To test the effect of intervention outcomes explored over time, linear mixed-model regression analysis with repeated measures over the three time points, T0, T1, T2, was used with 2 levels: measurements nested within individuals. Mixed-model regression accounts for autocorrelations and models data that includes missing cases. Statistical assumptions were tested regarding normal distribution, multicollinearity, homoscedasticity, and linear relationship between variables. The most suitable model for exploring group differences in intervention effects was explored by building nested models and comparing each model to the previous one using −2 Log Likelihood values to determine improvement of model fit. First, a model which only included fixed effects of exposure and time and an interaction term (exposure*time) was built. Second, a random intercept on level 2 (individual) was added to the first model. Third, a random slope on level 2 was added. Fourth, a three-level model was tested without a random slope, thus where model 2 was nested, where prisons were added as level 3. Significant improvement of model fit was achieved between the first and second model, whereas no significant improvement was achieved in the third or fourth model when a random slope and a third level were added. Therefore, the model used in the analysis of intervention effects included fixed effects of exposure and time and an interaction term (exposure*time) with a random intercept on level 2 (individual). Linear mixed-model regression was used for all outcomes except for contact frequency between parent and child where the intervention effect on the dichotomised variable displaying contact at least once per week or less than once a week was explored using mixed-model logistic regression. The sample size did not allow for any moderation analyses. Analyses were undertaken using the intention-to-treat (ITT) approach where all parents who consented to participation and provided data at baseline were included. This analysis is reported in the tables and in text. In addition, an analysis per protocol, using the same model as in the ITT analysis but comparing parents in the intervention group with attendance in all 10 intervention sessions to all participants who provided data in the control group, was undertaken but is reported in text only.

An a priori power calculation based on individuals and assuming equality between groups was conducted using the G*Power software [30]. The calculation was based on the desire to detect change in the primary outcome CPRS of a medium effect size (f^2^ = 0.15). Thus, with 85% power and a significance level of 5% a target number of 76 parents in total, including both study group, was identified to be included in the analysis.

Process data regarding fidelity and dose were analysed using descriptive statistics and is reported as means (SD), and proportions in text.

## 3. Results

### 3.1. Outcome evaluation

Six parents did not provide data at baseline and were therefore excluded from the analyses (Fig 1). These parents were equally distributed between the intervention and control groups and parental sex and represented four different prisons. Parental characteristics of the sample of 85 parents (intervention = 36, control = 49) that were included in the ITT analysis and group differences at baseline are presented in Table 1. No significant differences between the groups were found except for age, where the parents in the intervention group were older than parents in the control group. In the analysis per protocol, a total of 68 parents were included (intervention = 19, control = 49). Statistical assumptions for linear mixed-model regression were met. Unadjusted means for T1 and T2 are provided in S2 File.

**Table 1:** Descriptive characteristics of parents at baseline in intervention and control group.

	Total	Intervention	Control	*p*	*n*
	*n* = 85	*n* = 36	*n* = 49		
	Mean (SD)/%	Mean (SD)/%	Mean (SD)/%		
Parent sex–Father (%)	84.7	77.8	89.8	0.31	85
Parent age	36.38 (8.49)	33.81 (8.12)	38.31 (8.32)	0.02	84
Parents born outside Sweden (%)	25.9	36.1	18.4	0.67	85
Main source of income (%)					
Unemployment benefit	2.5	0	4.3	0.24	79
Social service allowance	12.7	9.4	14.9	0.47	79
Employment	67.1	68.8	66.0	0.79	79
Other	17.7	21.9	14.9	0.42	79
Education (%)					
<12 years (lower secondary school)	42.0	40.0	43.5	0.76	81
12 years (upper secondary school)	25.9	31.4	21.7	0.32	81
Vocational training post upper secondary school	19.8	20.0	19.6	0.96	81
University	12.3	8.6	15.2	0.37	81
Number of children <18 years	1.96 (1.26)	1.92 (1.16)	2.0 (1.34)	0.77	85
Co-habiting with at least one child prior to incarceration (%)	71.8	69.4	73.5	0.69	85
Co-habiting with at the other parent of one child prior to incarceration (%)	46.4	47.2	45.8	0.9	84
Custody of at least one child (%)	71.4	71.4	71.4	1.0	84
Allowed furlough (%)	28.2	26.5	30.6	0.68	83
Lived with at least one parent during own childhood	92.9	89.9	95.9	0.73	85
Rated importance of working with own parenting	8.96 (2.02)	9.17 (1.98)	8.81 (2.06)	0.43	83
Number of convictions	5.24 (8.69)	5.94 (8.75)	4.74 (8.71)	0.55	79
Length of current sentence (months)	56.89 (56.48)	61.81 (60.53)	53.29 (53.67)	0.5	85
Quality of child-parent relationship	4.43 (0.52)	4.42 (0.56)	4.43 (0.49)	0.92	62
Criminal attitude	2.34 (0.95)	2.29 (0.88)	2.38 (1.0)	0.68	85
Treatment interest	7.31 (3.14)	7.75 (2.97)	6.98 (3.26)	0.26	84
Child-parent contact ≥ per week (%)	62.0	54.5	67.3	0.24	79

p = significance level of difference between intervention and control group.

#### 3.1.1. Primary outcome

Of the 85 parents included in the overall analysis, 63 parents (intervention = 30, control = 33) met the inclusion criteria for the primary outcome and provided data at T0, corresponding to 69% of the entire study sample. There were no differences between parents with and without data on the primary outcome except for having lived together with a child prior to the incarceration which parents with data on the primary outcome had done to a higher extent.

**Fig 2:**
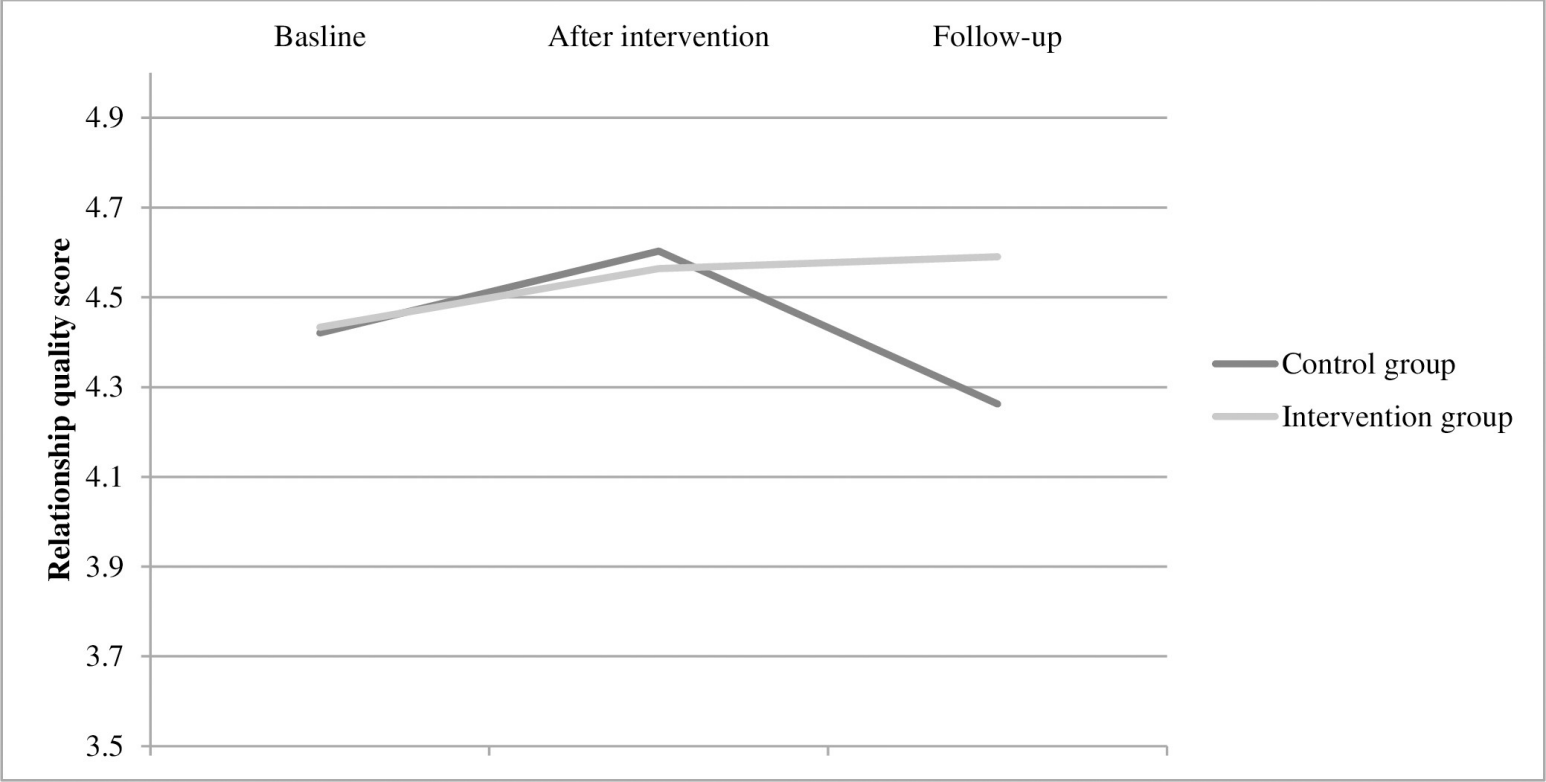
Mean scores for each group on relationship quality (CPRS) at each time point.

A significant group difference over time was found between groups regarding relationship quality between the parent and child (F = 4.11, df = 50.32, p = 0.022, Table 2), with the effect size of Cohen’s *f* = 0,342. This effect was explained by a significantly higher mean score for the intervention group compared to the control group at the follow-up measurement T2 (mean difference: 0.323, SE:0.161, p = 0,0497, Table 2, Fig 2), with the effect size of Cohen’s *f* = 0,226.

**Table 2:** Effect of intervention over time on primary and secondary outcomes in the intention-to-treat analysis. Estimates for the intervention group compared to the control group between each time point, and change over time.

Outcome	Mean difference (SE) on outcome for intervention group compared to control group at each time point						Change over time^§^ F (df)*	p	Between subject variance $\sigma_{u}^{2}$ (SE)	p
	Baseline		After intervention		Follow-up					
	Mean diff (SE)	p	Mean diff (SE)	p	Mean diff (SE)	p				
Quality of child-parent relationship	0.013 (0.136)	0.924	-0.037 (0.114)	0.748	**0.323 (0.161)**	**0,0497**	**4.110 (50.315)**	**0.022**	**0.139 (0.032)**	**0.00**
Criminal attitude	-0.088 (0.209)	0.673	-0.138 (0.198)	0.486	-0.409 (0.231)	0.079	2.305 (54.89)	0.109	**0.690 (0.120)**	**0.00**
Treatment interest	0.771 (0.704)	0.277	1.138 (0.710)	0.113	**1.965 (0.911)**	**0.036**	0.848 (68.596)	0.433	**4.800 (1.196)**	**0.00**

Control group = reference

^§^ Interaction: (group*time)

SE–standard error

Bold–p< 0.05

The analysis per protocol detected the same effects on group differences at T2, where the intervention group had a significantly higher mean score, with a stronger magnitude than in the ITT analysis (mean difference: 0.475, SE: 0.164, p = 0.004, Cohen’s *f* = 0,223).

#### 3.1.2. Secondary outcomes

**Fig 3:**
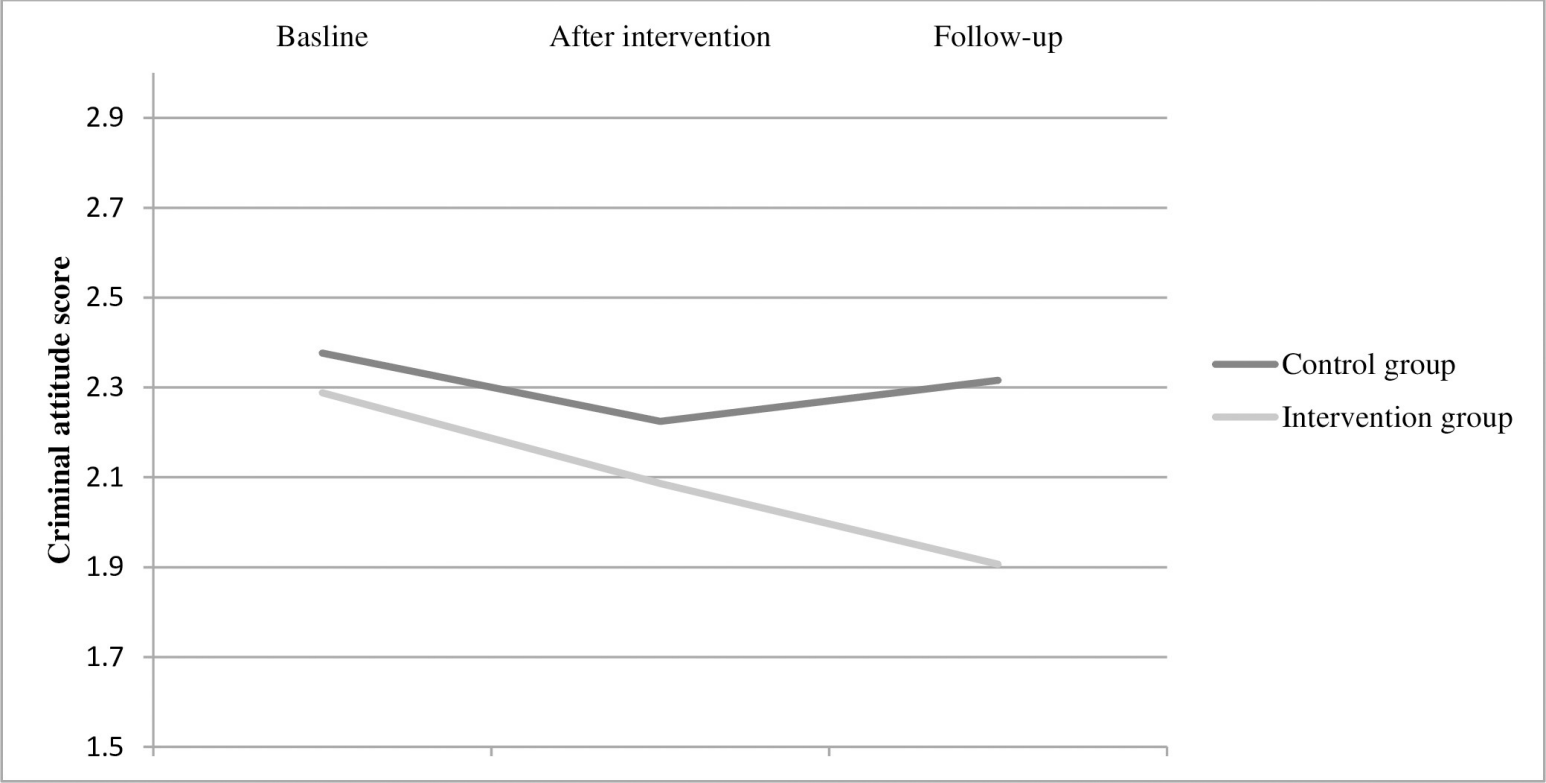
Mean scores for each group on criminal attitude at each time point.

*Criminal attitude*. No significant group difference over time was found between groups regarding criminal attitude (Table 2) and no significant difference was detected between the groups at either T1 or T2 (Table 2, Fig 3). In the analysis per protocol, a significant effect between groups over time was detected (F = 3.305, df = 76.442, p = 0.042, Cohen’s *f* = 0,241), explained by a significant effect between the groups at the follow-up measurement T2, where the intervention group had a lower mean score compared to the control group (mean difference: 0.798, SE: 0.259, p = 0.003, Cohen’s *f* = 0,283).

**Fig 4:**
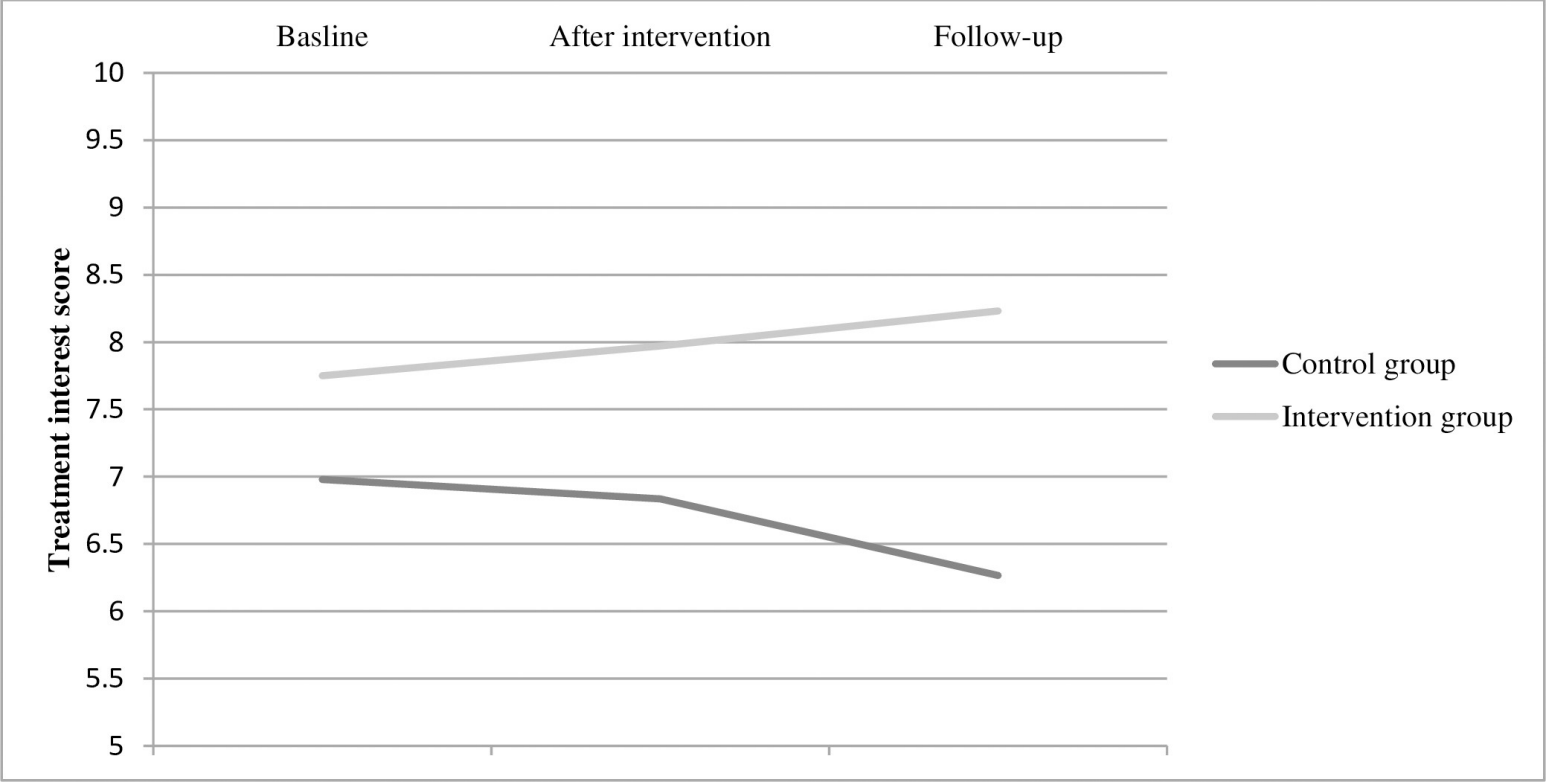
Mean scores for each group on interest in other treatment programmes at each time point.

*Interest in other treatment programmes*. No significant group difference over time was found regarding interest in other treatment programmes within the SPPS (Table 2). However, a significant mean difference between groups was detected at T2 where the intervention group had a 1,97 higher mean compared to the control group (p = 0,036) (Table 2, Fig 4), Cohen’s *f* = 0,255. In the analysis per protocol, the significant group difference at T2 was visible and in a similar magnitude (mean difference: 1.942), but did not reach statistical significance (p = 0.053).

*Contact frequency*. No significant group difference over time was found regarding the proportion of parents with contact with their child once per week or more in neither the ITT analysis (p = 1.0) nor the analysis per protocol (p = 1.0).

### 3.2. Process evaluation

#### 3.2.1. Observations of fidelity to intervention delivery regarding intervention knowledge, session structure and creating a supportive context, using the FIMP protocol

Ratings of the sessions rendered a mean value on the 9-point dimension Knowledge of 4.66 (SD: 5.1) for all prisons with minimum and maximum rates of 3.03 and 5.83 between the prisons. The mean value for all prisons on the dimension Structure was 4.68 (SD: 5.4) with minimum and maximum rates of 2.37 and 6.1 between the prisons. On the dimension Process the mean value for all prisons was 4.7 (SD: 1.52) with minimum and maximum rates of 2.24 and 6.53 between the prison. This means that the work conducted in the sessions was rated as acceptable with “adequate competence/adherence with some mistakes” (mean values between 4 and 6) mostly.

#### 3.2.2. Self-rated adherence to intervention delivery regarding core activities

Of the seven intervention groups that reported on fidelity using paper logs, all seven completed the 10 FOCS sessions and spent an average of 125 (SD: 18) minutes on each session. Of the 34 core activities in FOCS (two-four per session), 25 (74%) were completed according to the manual or with one of the seven groups deviating partly from the manual, and 9 (26%) sessions where two to four of the groups deviated partly from the manual. On four occasions, a core activity was not delivered at all (different activities by different prisons). When activities were only partly completed, they were instead given as home assignments to the participants at times. The majority of the group sessions were conducted by the original two group leaders, but in two groups, an alternative constellation, where one group leader was substituted in a session, performed the FOCS sessions on nine occasions.

#### 3.2.3. Dose received

Seven of the eight intervention FOCS group reported participation rates (33 participants of the 36 in total in the intervention group). These participants had an average participation in 8 FOCS sessions (SD: 2.9) where 19 had participated in all ten sessions and six had participated in less than five sessions.

## 4. Discussion

This pragmatic controlled study which was carried out with incarcerated mothers and fathers in prisons in Sweden with the aim to support positive parenting to influence healthy child development showed significant effects on quality in the relationship between parent and child and indicated effects on criminal attitude and interest in participating in additional treatment programmes during the time in prison. These are encouraging results for both the children, who face great risk of poor-health and marginalisation across development, and for the parents who may be supported in changing their criminal behaviour in addition to engaging in more positive parenting.

The results of this study showed a significant effect of intervention on the primary outcome quality in relationship between child and parent in both the ITT and per protocol analyses, which confirms the hypothesis of the study, although the hypothesised primary end point was T1, after the intervention, when the effects were in fact identified at the follow-up measurement T2. The beneficial effects on the primary outcome align with previous evaluations of interventions for incarcerated parents on the international arena [16, 18]. A meta-analysis which encompassed 10 evaluations of such interventions using randomised and quasi-experimental study designs where intervention was compared to waitlist, no intervention, or treatment as usual, concluded a pooled intervention effect of a small (SMD = 0.27, 95% CI 0.02, 0.51) effect size on quality of relationship between parent and child [18]. Similarly, a systematic review investigating the effects of parenting interventions for incarcerated parents, but which only included mothers, concluded the same type of intervention effects on relationship quality [16]. Thus, the findings of the current study align with results from the international arena and adds new insight regarding beneficial effects of parenting interventions for incarcerated parents in the quality of the relationship between parent and child, focusing on warmth, in a Northern European context. Warmth in the relationship between parent and child can be an important factor in the promotion of child healthy development. Early studies have shown that authoritative parenting, which includes involved, warm and at the same time firm, consistent, and developmentally appropriate parenting is positively associated with adolescent psychological well-being such as less depression, anxiety, and antisocial behaviors [31]. Further, retrospectively reported parental warmth predicted flourishing in terms of emotional, psychological, and social wellbeing ten years later in mid-life [32], whereas in a 20-year longitudinal study retrospectively reported higher levels of parental warmth were related to less negative- and more positive affect in adulthood [33]. In addition, whereas The Dunedin Multidisciplinary Health and Development birth cohort, which has been running for 50 years, has found that low adolescent self-esteem was related to negative well-being and criminality ten years later [34], another, 17-year longitudinal study of adolescents [35] reported that parent-child relationship was a significant predictor of global adolescent self-esteem. Thus, increased warmth in the relationship between parent and child may influence important aspects in healthy development in these children in a longer term and should be further investigated in future research. Also, observational measurements should be considered in future studies, to replicate and strengthen the findings of this study where self-reported questionnaires were used. Furthermore, the number of mothers incarcerated in Swedish prisons is small and the sample in this study did not allow for sub-group analyses, However, future studies should consider investigating differences between mothers and fathers with regards to effectiveness of a prison parenting programme. Such investigation could guide further development of sex specific prison parenting interventions.

The results of the intervention effects on the secondary outcome are also in line with the hypothesised results of the study, although weaker than the results for the primary outcome. These outcomes comprise interest in participating in additional treatment during the incarceration, and criminal attitude, which have been less studied in previous trials that investigate the effectiveness of parenting interventions for incarcerated parents. This restricts the possibility to compare these findings with previous studies, but where the findings of this study indicate the possibility of effects on outcomes related to criminal attitude and rehabilitation as a results of parenting interventions for incarcerated parents. Intervention effects at the follow-up measurement were found for improved criminal attitude when comparing parents in the intervention group who participated in all 10 sessions with parents in the control group, and for interest in participating in treatment programmes in the analysis using the ITT approach. Thus, the intervention, with a focus on enhancing the parenting role in terms of recognition of parenting responsibility and positive practices, increasing knowledge and understanding of children’s development and needs, seemed to have an influence on aspects that are related to attitudes towards criminality, where a decrease in criminal attitude resonates with a more prosocial attitude, and where interest in treatment programmes in prison conveys a motivation to change criminal behaviour as treatment interventions in prison are focusing on supporting behaviour change towards a non-criminal lifestyle [22]. Although few prior studies have investigated these outcomes as an effect of parenting interventions in prison, research has hypothesized links between parenting interventions in prison and a decrease in recidivism, e.g., through the building of strong social bonds [20], or through stronger identifications with the role as a parent [36]. In a study from the US, Burraston & Eddy [20] propose that a parenting intervention in prison may link strong social bonds [37], critical life events such as having a child [38, 39], and turning points such as incarceration resulting in contemplation and concerns for the child [40], with reduced recidivism [20]. The study found evidence that a stronger social bond, as in living with a child prior to incarceration, predicted a decrease in recidivism in the control group, where the parenting intervention may be especially effective for parents who did not live with their child prior to the incarceration [20]. In the Northern European setting, Hansen et al propose that parenting interventions for incarcerated fathers in Norway may lead to improved criminal attitude through a stronger identification with the role as a parent [36]. Qualitative findings with interviews before and after the interventions indicated prosocial changes in criminal attitudes among the fathers who saw that their criminal lifestyle was conflicting with their responsibilities as engaged fathers for their children [36]. The findings of the current study support the suggestion that parenting interventions for parents in prison may have beneficial influences on outcomes related to criminal attitudes. Further research should investigate the chain between improved parenting and a decrease in criminality further, e.g., through study designs that run over a longer period of time and include outcomes on recidivism. Such long-term outcomes on both parent and child delinquency have been described in the programme theory of FOCS, published in the study protocol [24] which can serve as an example.

The secondary outcome child-parent contact frequency did not render any effect of intervention and is also difficult to interpret. The contact that the parent is allowed to have with a child is dependent on the regulations in the prison, where different security levels have different regulations for telephone contact, visits, and leave. In future studies, a measurement that better reflects the contact between child and parent should be considered.

Although the results of this trial have shown beneficial intervention effects on both primary and secondary outcomes, a greater magnitude and more consistent effects over both the ITT and per protocol analyses would have been desired regarding the secondary outcomes. Therefore, revisions of the intervention activities should be considered to possibly increase intervention effectiveness. Importantly, the results of process evaluations of the FOCS intervention which are reported in a mixed-methods study found that delivering group leaders, and managers responsible for the implementation of FOCS in prison called for further development and revisions of FOCS to make the intervention adaptable to the different needs of the parents who participate, e.g., extend the intervention to include additional themes, adapt certain themes to cater to gender differences, include individual sessions in addition to group sessions to facilitate discussion regarding sensitive topics, and more in-depth knowledge on parenting practices [41]. In the general population, parenting programmes that are based on cognitive behavioural therapy have shown beneficial effects [42]. Programmes that have proven effective when conducted with parents in prisons specifically have included practice-oriented elements such as activities focusing on altering a behaviour or parenting skills training [16]. The most extensive evaluation on effects of a parenting intervention for parents in prison was conducted with the Parenting Inside Out intervention which is based on Parent Management Training Oregon (PMTO) [43] and includes knowledge and skills training including practice exercises, role plays, and homework which provides the parent with opportunity to practice in-between sessions [21]. The FOCS intervention is based on developmental psychology, attachment theory, social cognitive theory, and the Convention on the Rights of the Child, and includes material and activities that focus on group discussion and reflection on child issues and parenting but does not include activities where parents practice parenting behavior such as skill training exercises or role-play. The theoretical foundations would permit the inclusion of additional components such as role play where parents would have the possibility to practice parenting skills which could enhance the effects of the intervention. Importantly, skills training and behaviour change requires work over a longer period of time, especially if skills training of behaviours in focus, as parenting, is limited due to limited child-parent contact. Therefore, a chain of interventions that focus on parenting and child-parent interaction during the period of incarceration should be considered.

Yet another factor which can be increased in order to improve intervention effects on outcomes is the fidelity to intervention delivery. High fidelity of intervention delivery of parenting programmes has been linked to higher intervention effects on outcomes [44, 45]. In this study, group leaders self-reported that they had performed all 10 sessions and that they had performed 74% according to how they were described in the manual, which can be considered high. The researcher-ratings of group leader delivery “knowledge”,” structure”, and “process” in the sessions resulted in mean values for all prisons on which corresponded to delivery on a level which is “acceptable with adequate competence/adherence with some mistakes”, and where the range revealed that some prisons were on a level corresponding to delivery that “needs work–insufficient adherence”. These results convey that group leaders overall conduct the sessions and activities that are stated in the manual, but that the mode of delivery could be improved. This is further supported by the mixed-method process evaluation of FOCS where group leaders identified that it was challenging to deliver FOCS, which touches on very sensitive topics, with parents in prison who often have very heterogeneous and troublesome backgrounds, and complex needs [41]. To handle these challenges, both the group leaders themselves and the responsible managers called for more in-depth competence to handle the sensitive topics, and interaction in the group, which could improve fidelity to intervention delivery. In addition, the process evaluation showed that the structure of the FOCS manual could be improved to further guide the group leader in how to deliver the intervention activities with high fidelity. Thus, it seems that increased training, or competence, and revisions of the intervention manual could enhance the possibility for group leaders to deliver FOCS with high fidelity.

### 4.1. Strengths and limitations

Strengths of this study are severalfold and comprise the broad setting within the Swedish context where prisons on all security levels of the SPPS including low, medium and high levels participated in the trial. Furthermore, both mothers and fathers participated, whereas most previous evaluations of intervention for incarcerated parents have included only one sex, either mothers or fathers, (see e.g., [16, 18]). Furthermore, this trial included a follow-up measurement. Such measurements are of essence for understanding the sustainability of intervention effects but can be difficult to accomplish in the normal population and even more difficult in populations which are only present in the prison setting during a specific period of time, i.e., when serving the sentence. Yet another strength is the use of well-established and valid questionnaires, and the use of objectively rated measures for fidelity of intervention delivery with high inter-rater reliability using a well-established instrument [29]. Fidelity to intervention delivery is of essence to monitor in order to be able to make correct interpretations of intervention effects [46]. There are several ways of measuring fidelity to delivery, where self-report of completed sessions or activities is common and inexpensive, but can be less reliable, whereas the type of objective ratings using trained raters and validated instruments, as used in this study, is time-consuming, expensive, but often more reliable and therefore increase the trustworthiness of the interpretation of the intervention effects. Limitations comprise the sample size, and the uneven numbers of participants in each group, especially regarding the per protocol analysis. The sample included in the analysis of the primary outcome entailed a total of 63 parents whereas the power calculation was set for 76 parents. Although a beneficial effect of intervention was detected, additional effects may have gone unnoticed in this trial due to the limited sample size. In addition, the small sample size prohibited the possibility of moderation analyses which could have provided important information on e.g., sub-group for whom the intervention was more of less effective. In addition, although a follow-up measurement was included in the study, the measurement time points can be considered close in time and a follow-up measurement more distant in time may provide more sufficient opportunity for change. Also, the latter part of the study was conducted during the covid pandemic, which made data collection more difficult and may have resulted in missing data. Furthermore, the lack of randomisation may have resulted in systematic differences between the intervention and control groups. However, no such differences, except for parents’ age were visible in the between group-test at baseline, but differences that have not been measured may exist. A further limitation comprises the use of measurements which reflect the parent-perspective of quality in the relationship with the child only. Here, more objective measures, e.g., video recorded or real-life observations of child-parent interaction, could shed light on actual parenting behaviour as well as on other aspects of the relationship between the child and parent. In addition, lack of information regarding type of conviction comprises a limitation to the study. Furthermore, the measurement regarding child-parent contact frequency turned out to be difficult to use, which comprises a limitation. In addition, the non-incarcerated caregiver is an important link between the incarcerated parent and the child. Therefore, the inclusion of measurements that capture the cooperation between the incarcerated parent and the other caregiver could be useful to include in future evaluations.

### 4.2. Conclusion

The FOCS intervention had beneficial effects on relationship quality, and potential to influence criminal attitude and interest in other treatment intervention. This is the first scientific evaluation of a parenting intervention for incarcerated parents in a Northern European setting and the findings show that a parenting intervention for incarcerated parents has the potential to influence both parenting outcomes but also outcomes related to a criminal lifestyle. These are encouraging results for both the children who face great risk for poor health and marginalisation, and for the parents who may be supported in changing their criminal behaviour. Future studies should investigate intervention effectiveness on long-term outcomes related to both child health and parental recidivism and could also be expanded to probation and jail settings, as well as other Northern European countries. Further development of intervention components could be made which focus on practicing parenting skills with the hypothesis that such developments could increase intervention effectiveness.

## Supporting information

S1 File TREND checklist. (PDF)

S2 File Unadjusted means. (PDF)

S3 File Ethical application. (PDF)

## List of abbreviations

FIMP: The Fidelity of Implementation Rating System
FOCS: For Our Children’s Sake
ITT: Intention to treat
SPPS: The Swedish Prison and Probation Service
