# Supplementary material for: Effects of a parental support intervention for parents in prison on child-parent relationship and criminal attitude—The For Our Children’s Sake pragmatic controlled study
Source: PLoS One. 2023 Mar 23;18(3):e0283177. doi: 10.1371/journal.pone.0283177 (PMC10035849; doi:10.1371/journal.pone.0283177)
Supplement: S2 File — (PDF) [file pone.0283177.s002.pdf]

Supplementary information 2. Unadjusted means and percentage

Outcome	Unadjusted means (SD)/% total and per group at T1 and T2											
	After intervention (T1)						Follow-up (T2)					
	n	Total	n	Int	n	Cont	n	Total	n	Int	n	cont
Quality of child-parent relationship (mean/SD)	40	4.58 (0.4)	22	4.55 (0.35)	18	4.61 (0.46)	29	4.49 (0.52)	16	4.54 (0.52)	13	4.43 (0.53)
Criminal attitude (mean/SD)	51	2.18 (0.83)	24	2.05 (0.67)	27	2.29 (0.95)	39	2.13 (0.96)	20	1.94 (0.88)	19	2.33 (1.02
Treatment interest (mean/SD)	50	7.36 (2.94)	23	8.17 (2.33)	27	6.67 (3.26)	39	7.31 (3.13	20	8.15 (2.66)	19	6.42 (3.41)
Child-parent contact ≥ per week (%)	45	66.7	18	61.1	27	70.4	35	77.1	17	70.6	18	83.3
