# Supplementary material for: Effects of a parental support intervention for parents in prison on child-parent relationship and criminal attitude—The For Our Children’s Sake pragmatic controlled study
Source: PLoS One. 2023 Mar 23;18(3):e0283177. doi: 10.1371/journal.pone.0283177 (PMC10035849; doi:10.1371/journal.pone.0283177)
Supplement: S3 File — (PDF) [file pone.0283177.s003.pdf]

Ethical application

Title

Parental support in prison - evaluation of a parental support intervention in correctional services to promote positive parenting among parents in prison

Aim and research questions

What is the scientific purpose of the project?

International research shows that parental support interventions for incarcerated parents can have a positive impact on parenting, the relationship between children and parents and the children's well-being, as well as to some extent on recidivism, but no scientific evaluations have been carried out on the parental support intervention which is currently offered in the Swedish Prison and Probation Service (SPPS). The overall aim of this project is to find out if the parental support effort that is currently offered to incarcerated parents in prisons in Sweden, "For our children's sake" (FOCS), has an effect on outcomes linked to parenting and attitude to crime. The project also includes monitoring of the implementation process of the intervention as well as qualitative studies to investigate potential revisions of the intervention to enable increased positive effects on the parents. Objectives of the project thus relate to two parts:

1. Evaluation of the effects of the FOCS intervention on outcomes linked to parenting, frequency of contact between child and incarcerated parent, attitude to criminality and interest in participating in treatment of the incarcerated parent through a controlled study
2. Evaluation of the implementation process of the FOCS intervention through mixed-methods

What are the scientific questions?

- What are the effects of the FOCS intervention on parenting, attitude to criminality and propensity to participate in treatment of the detained parent?
- With what adherence was the intervention carried out and what hindering and facilitating factors exist for the implementation of the FOCS intervention?
- Are the effects on quality in the child-parent relationship mediated by the parent's attitude toward parenting and parental self-efficacy?
- What is the view of the incarcerated parents themselves, group leaders and prison managers on the parenting of incarcerated parents?
- What barriers and facilitators emerge during the FOCS intervention according to detained parents, group leaders and managers?
- What psychometric properties does the instrument AAPI2 have in the Swedish context?

Methods

Describe the method including the procedure, technique or treatment.

Outcome measures obtained before intervention T0, after intervention T1 and at three months of follow-up T2. Mediators are also measured in intermediate measurement TM (after group meeting 6). Survey responded to by parents in custody

<u>Primary outcome:</u>

Quality of the relationship with the child/nen with the internationally validated and well-used Child Parent Relationship Scale CPRS (Pianta, 1992) where subscale measuring closeness is used (7 items). The scale has been translated into Swedish.

Secondary outcomes

- Attitude to criminality - measured by the construct antisocial intent in the validated soul assessment instrument Measures of Criminal Attitudes and Associates (MCAA) validated in the Swedish context (Bäckström & Björklund 2008).
- Interest in participating in other treatment within the Prison and Probation Service - is measured self-reported with an item.
- Frequency of contact with the child - measured by the parent's self-reported type of contact (letter/phone/visit etc) as well as frequency.

Mediators

- Attitude to parenting is measured by the internationally validated survey The Adult-Adolescent Parenting Inventory 2 (AAPI-2) (Bavolek &Keene 2001) - where 14 translated items are used. Items are pilot-tested with approximately 5 incarcerated parents to ensure that they are understandable and relevant adapted to the Swedish context. Also measured at TM.
- Parental self-efficacy measured with specifically designed questionnaire with a maximum of 10 items pilot-tested as above. Also measured at TM.

Demographic questions asked only in the baseline measurement. Questions to incarcerated parents include age, country of birth, sentence time, number of sentences in total, number of children, contact with their children who are sent as moderators in the analysis of outcome measures. Questions to managers and group leaders relate to age, gender, education and professional experiences. These are then intended to be used as moderators in the analysis of outcome measures.

Measures related to the intervention process are obtained during and after intervention

- The group leader notes during the implementation phase:
  - Dose - how many of the total number of group meetings each participant has attended
  - Reach - how many inmates in the institution/department meet the inclusion criteria, which is then compared to the number of participants in the group
  - Fidelity - each meeting is audio recorded by the group leader and the group leader fills in short logs after each meeting regarding the implementation of central parts of the meeting, adaptation of the meeting, reception in the group based on structured form developed by the researchers.
- Post-intervention interviews take place with parents (about 15), group leaders (about 10) and managers (about 5) in the intervention group per recorded phone call that is then transcribed verbatim where specific, identifying information (e.g., names, locations) is removed. The interviews are conducted by researchers and focus on discussion themes regarding the experience of participation/implementation of FOCS, adaptations, meeting the needs of inmates, needs for implementation, maintenance, revision of the intervention, and the view of the parenting of the own/that of incarcerated parents.
- Questionnaire to group leaders, managers and parents at T1 which, for managers and group leaders, includes 17 items and for parents 9 items about experiencing the needs of the target group (parents), as well as the capacity to carry out the intervention at the authority, institutional and individual level. Items are adapted based on established instruments (Fernandez ME et al 2018, Kegler et al 2018) and will be pilot-tested on 2 managers and 2 team leaders

Describe in what way the method differs from clinical routine or the treatment as ususal

FOCS is part of the SPPS's activities at prisons and is currently carried out in a number of Swedish prisons. In this way, the intervention already constitutes the usual routine for how parental support is provided in Swedish prisons. The group leaders at the prisons have received training in conduting FOCS. Training (3+2 days) of new group leaders takes place on a continuous basis and is carried out under structured auspices by the Prison and Probation Service where special group leader trainers lead and follow up training with the group leaders. Implementation and administration of the parent groups is done by group leaders in the respective prison, usually correctional officers who conduct groups as part of their service. This project is thus carried out on an ordinary, existing activity.

Describe previous experience (own and/or others') of the procedure, technique or treatment used

The methods applied in the present research project are scientifically proven and established within the research community. Responsible researchers have a combined experience and knowledge of applying relevant quantitative and qualitative data collection methods that have previously resulted in a number of scientific publications. The researchers have good knowledge and experience of working with questionnaires, interviews, and have experience of working in correctional institutions and the specific conditions that prevail in the prison environment. A project group with contact persons at different levels within the SPPS will, together with the researchers, discuss issues that arise in connection with the implementation of the project in order for it to be implemented in such a balanced way at the various institutions. The parental initiative FOCS is already carried out as structured activities for clients in correctional institutions in Sweden. The effort focuses on positive messages around parenting and children's needs. Similar types of parental interventions for clients serving prison sentences exist several countries worldwide. The specific initiative FOCS is also used in Switzerland and has formed the basis and inspiration for the parental efforts conducted within the Danish and Norwegian prison services.

Time plan

Expected project start date

August 26th, 2019

Expected project completion date

January 1st, 2022

Timeline for the different parts of the project

Data collection Baseline T0: September Intermediate measurement TM circa November 2019 Post-measurement T1: January 2020 Follow-up measurement T2: April 2020 Interviews April - June 2020 Data management, analysis and reporting: August 2020-December 2021

Data collection

Describe data collection and nature of the data

<u>Paper questionnaire to incarcerated parents answered at T0, T1 and T2. Mediators also at TM.</u>

Quality of the relationship with the child is measured with the Child Parent Relationship Scale. If there are several children, one form is provided per child, adapted to the age of the child. This is determined by the detained parent's response to the number of children and ages upon consent

Attitude to criminality – measured with Measures of Criminal Attitudes and Associates

Frequency of contact with the child - the parent self-reports the type of contact, as well as frequency. This is indicated per child.

Interest in participating in other treatment within the Prison and Probation Service - measured self-reported with an item.

Attitude to parenting – measured with The Adult-Adolescent Parenting Inventory 2. Also measured at TM.

Parental self-efficacy to performing positive parenting behaviour- with study-specific designed questionnaire. Also measured at TM.

Demographic questions are asked at baseline measurement only.

Questions regarding implementation factors are asked to T1 only.

Questionnaires for all participating parents in a prison are sent by post to the prison in a package including sealed envelope. Each sealed envelope has the participant's first name on it and contains the questionnaire, with code number only, as well as a blank envelope in which the participant puts the completed questionnaire and then seals. All blank envelopes with participant repsonses are collected by prison staff and sent in joint package by registered mail or handed over in person between staff and researchers when distance and travel allow. The envelope with the participant's first name is discarded.

<u>Questionnaire to group leaders, managers (web) and incarcerated parents (paper) measured at T1</u>
These measure factors of importance for successful implementation of intervention as well as the needs of the target group. Parents in connection with T1 (see above) and staff via web (see below).

Mangers and group leaders fill in a number of background questions regarding, age, gender, education and professional experience.

The online surveys for staff are made available via web link to KI-survey, Karolinska Institutet's web solution for surveys that meet requirements for secure data management. The web link is distributed to the participant's specified email address where the link leads directly to the survey on Karolinska Institutet's server.

<u>Interviews with incarcerated parents, group leaders and managers</u>

- Interviews take place with parents (about 15), group leaders (about 10) and managers (about 5) by phone where the call is recorded and then transcribed verbatim, but where specific, identifying information (e.g. names, locations) is removed. The interviews are conducted by researchers and focus on discussion themes regarding the experience of participation/implementation of FOCS, adaptations, meeting the needs of inmates, needs for implementation, maintenance, revision of the effort, and the view of the parenting of the own/incarcerated parents.
- The participant is informed and specifically consents to participation in the interview.

<u>Measures related to the implementation process</u>

The group leader notes during the implementation phase:

- Dose - participant attendance is recorded by group leaders (with separate participant code, not the same code as survey)

- Reach - how many inmates in the prison that meet the inclusion criteria, which is then compared with the number of participants in the group, is noted by the group leader
- Fidelity - each meeting is audio recorded by group leaders and group leaders fill in short logs after each meeting.

Data regarding the implementation process will be transferred from the Prison and Probation Service/group leader to the responsible researcher via a shared folder in a cloud solution secured by Karolinska Institutet (owncloud) where group leaders have access to the cloud through individual passwords and can only upload material. Group leaders delete the audio file once it's uploaded to owncloud. Group leaders follow a structured manual/checklist for handling audio files from recording to uploading and deleting.

Describe the statistical basis for the pre-study population/survey data size

The study will be conducted as a controlled study. Sample size has been calculated at the individual level where the calculation is based on the primary outcome, where previous studies of parental support for parents in prison have shown that positive changes in parenting with moderate effect size can be achieved as a result of intervention (Scudder et al 2014, Menting et al 2014). In this study, we wish to increase positive parenting, relative to the control group, with an effect size of 0.5. With 80% power and 5% significance level, it is then desirable to have a total of 198 incarcerated parents, who are evenly divided between intervention and control groups.

Interviews are conducted with parents (approximately 15), group leaders (approximately 10) and managers (approximately 5) based on the qualitative selection strategy appropriate selection with maximum variation in terms of safety class in prison, frequency of contact with children (applies to parent only), gender, country of birth (applies to parent only), experience of leading group (only applies to group leader).

How will the investigation procedures be documented?

<u>Paper questionnaire for incarcerated parents</u>

Questionnaires for all participating parents in a prison are sent by post to the prison in a package including sealed envelope. Each sealed envelope has the participant's first name on it and contains the questionnaire, with code number only, as well as a blank envelope in which the participant puts the completed questionnaire and then seals. All blank envelopes with participant responses are collected by prison staff and sent in a joint package by registered mail or handed over in person between staff and researchers when distance and travel allow. The envelope with the participant's first name is discarded. The questionnaires are then pseudonymised and transferred to digital file by the researchers.

<u>Interviews with incarcerated parents, group leaders and managers</u>

Interviews are audio recorded and transcribed verbatim. Audio and text files are stored on KI's server or on an external hard drive in a fireproof cabinet.

<u>Measures related to the implementation process</u>

Fidelity - each meeting is audio recorded by group leaders and group leaders fill in logs after each meeting based on structured form developed by the researchers that is distributed via web link to survey. The online surveys are made available via web link to KI-survey, Karolinska Institutet's web solution for surveys that meet requirements for secure data management. The web link is distributed to the participant's specified e-mail address where the link leads directly to the survey on Karolinska Institutet's server. Online surveys are downloaded from KI-survey and stored as a data file on KI's

server. Other data regarding the intervention process will be transferred from the Prison and Probation Service/group leader to the responsible researcher via a shared folder in a cloud solution secured by Karolinska Institutet (owncloud) where group leaders have access to the cloud through individual passwords and can only upload material. Data is downloaded from owncloud and stored as a data file onKI's server and/or on an external hard drive in a fireproof cabinet.

How will the collected data be handled and stored?

All names and personal details of participants will be replaced with a code number for the purpose of de-identifying participating persons. Code lists and lists names and contact details are stored separately locked in filing cabinets. All data, including audio recordings from group meetings and interviews, is stored on a server at Karolinska Institutet with access control (only for the project group), backup and virus protection. Paper questionnaires are stored in a locked metal cabinet. The material processed and analyzed is guaranteed the necessary protection of confidentiality. During the project, the principal investigator and participating researchers have access to the material. Collected material will be archived after the end of the project in accordance with current rules. Notification of personal data processing will be made to Karolinska Institutet. The study is also reported to the International Standard Randomised Controlled Trial Number (ISRCTN) registry.

Electronic data collected via KI's digital survey solution KI-survey and transmitted via owncloud will be handled in accordance with the Swedish Data Protection Authority's regulations. Data collected via KI-survey and owncloud will be downloaded and stored as data files on KI's server and/or on external hard drive in a fireproof cabinet.

Ethical Considerations

What risks can participation entail for the research subjects included in the research project?

The FOCS intervention is itself designed to increase parents' positive parenting in order to promote children's healthy development. The initiative is already implemented as part of the regular activities at correctional institutions in Sweden and is carried out as a group activity. Participants in the group may, by gaining an increased insight into their parenting and the needs of the children during the group meetings, experience discomfort in cases where they view their previous parenting as deficient based on the newly gained insights during the group meetings. The control group's implementation of FOCS after the follow-up measurement is dependent on the specific institution's operational planning, which in itself is dependent on human resources and core activities at the institution, the timely planning of group implementation may vary between institutions, which in itself can possibly be frustrating for control group participants, but is entirely in accordance with the activities' own planning and not linked to the implementation of the project. As part of this research project, participants will complete questionnaires regarding their attitude to parenting, parental self-efficacy, the quality of the relationship with their children and the frequency of contact with the children. All of these measures can remind the parent of how the time in prison negatively affects the relationship with the child/ren and thus cause discomfort in the parent in connection with these reminders. It is also important to highlight the possibility that the fact that the parent is in custody may affect the feeling of voluntariness in participation. It is therefore important to stress the importance of one's own voluntary decision in the context of informed consent during the recruitment of participants. All information will be provided in writing, but as much as possible also orally to facilitate understanding for parents with, for example, limited knowledge of Swedish.

What benefit can participation bring to the research subjects involved in the research project?

Studies show that children of incarcerated parents have a poorer well-being, compared to children without incarcerated parents. These children also have a higher risk of their own crime later in life, with a dose-response correlation between the number of times the parent served a sentence and an increased risk of their own crime. For the incarcerated parent, separation from the child(s) as a result of the prison sentence can lead to stress, despair and powerlessness in relation to parenthood. Positive parenting is an important factor for children's healthy development. Incarcerated parents may have difficulties in conducting positive parenting due to circumstances and conditions that negatively affect parenthood, such as drug use, poverty or lack of experience of positive parenting in their own childhood. Internationally developed interventions for parents in prison have been shown to influence outcomes related to parenting such as positive interaction between parents and children, knowledge of parenting, empathy, stress in parenting, increased contact between parent and child, and active parenting. Through participation in For the Sake of Our Children, detained parents receive support for positive parenting, which in the fief can lead to a better and closer relationship with their children. Group participation also gives the parent the chance to actively work on their parenting and discuss with others in a group, which should be able to mitigate the negative feelings and thoughts about how their own punishment affects the family and children. Participating in the research study gives the parent the opportunity to contribute to the generation of knowledge about parental support interventions for detained parents, which is beneficial for the individual parent, but also for future parents who participate in the intervention, in the current or revised form, in the future.

Evaluate the relationship between the risks and benefits of the project

The risks are assessed as very small in relation to the benefits the project generates in the form of increased knowledge about how parental support work for parents and children and what needs for revision of the intervention exist to create positive effects as a result of the intervention. The project will also generate knowledge about parental support in correctional care, which is of great importance for the future work within the authority in supporting detained parents in their parenting, which is of benefit not only to the group participating in this project, but also parents who in the future will be reached by parental support efforts in Swedish correctional care.

Describe how the project has been designed to minimize the risks for the research subjects

From an ethical perspective, it is important to highlight the possibility that the fact that the parent is in custody may affect the sense of voluntariness in participation. It is therefore important to stress the importance of one's own voluntary decision in the context of informed consent during the recruitment of participants. All information will be provided in writing, but as much as possible also orally to facilitate understanding for parents with, for example, limited knowledge of Swedish. Research involving children is always a basis for careful ethical considerations because children do not themselves consent but depend on others to make decisions for them. Since this evaluation focuses on parental support, children are not actively affected. However, it may be the case that children of parents serving sentences in prison may have experiences of traumatizing experiences during their upbringing based on, for example, their parents' drug abuse, violence or threats of violence. It is therefore of paramount importance that no child is forced to have contact with an unsuitable parent. For this reason, those parents in detention who have committed a crime in which the child is a victim or who have committed a violent crime against the child's other parent will be excluded from this evaluation. This type of information is available to correctional team leaders who will ensure that no group participant who committed crimes as described above is invited to participate in the evaluation. The instruments used for the different dimensions within the project

have been carefully selected so that they are as suitable for the operation as possible, as well as as valid and comparable dimensions as possible, but at the same time to arouse as little discomfort as possible for the parents

Identify and specify whether any ethical problems (disadvantages/advantages) may arise in a broader perspective through the research project

The project aims to evaluate whether the intervention positively affects parenting among detained parents in Swedish correctional services. International studies have shown that parental support efforts in prisons can affect parenting, and have also shown positive effects on children's behaviors and reduced recidivism in crime among the parent. This project evaluates an already ongoing intervention within the Swedish prison service that has not yet been scientifically evaluated. The benefits of this project are thus to generate scientifically reliable knowledge about how the intervention affects incarcerated parents and will form the basis for future parental support efforts as well as any revisions of the same within the Swedish penitentiary. In addition to this, it should also be taken into account that these children belong to a risk group for low well-being and self-crime, this can potentially reduce the risk of the child's later crime in the longer term. In addition to this, the children's poor well-being and later crime represent major societal costs, for example through health care, schools, resources, crime victims and the justice system including the Prison and Probation Service. Thus, in the event of positive effects on outcomes, several societal costs can be reduced.

Research subjects

How is the selection of research subjects made?

Step 1. All prisons with trained group leaders in FOCS are invited to participate in the evaluation. Eligible prisons are identified by group leader trainers within the SPPS. Step 2. At the prison that accept to be included in the evaluation, group leaders in the intervention group, who will carry out FOCS, recruit group participants according to the usual procedure where all parents who are interested in participating are interviewed individually, whereupon the group leaders put together a suitable group based on reasoning about group dynamics, background and needs of group participants. Of the recruited group participants, group leaders then identify and invite all those who meet the inclusion criteria to participate in the evaluation. Group leaders follow a checklist of inclusion criteria and are instructed by researchers to ensure, as a first step, that there are no contact bans for children and/or the other parent and that no violent crime has been committed against the other parent before the remaining inclusion criteria are reconciled and the invitation to participate in the evaluation is carried out. Group leaders at institutions within the control group follow the same procedure, but with the clear difference in information that all participants will fill in the measurements (participate in the evaluation) before implementing the parental support intervention and that the intervention may be delayed, but information about when in time the intervention is planned at the specific institution based on existing operational planning.

How many research subjects will be included in the research project?

Parents in prison: in terms of outcome measures, the goal is to include a total of 198 detained parents in interviews, the goal is to include parents (about 15), group leaders (about 10) and managers (about 5).

What selection criteria will be used for inclusion?

At least one child aged 0-18 years - parents who have children aged 0-2 and 13-18 years will not be included in the primary outcome measure. These parents then respond only to the mediating measures regarding self-efficacy and attitude toward parenting. This is because the primary outcome

measure is not adapted to for children 12 years old. Otherwise, the parents are included in all other measures and sub-studies the parents have the legal right to be in contact with the child The parents have not committed crimes against the child or violent crimes against the other parent have sufficient knowledge of the Swedish language in such a way that they can participate in the group, according to the group leader's assessment have contact with the child/ n. In cases where the detained parent does not have or has not had contact with his/her children, they may be included in mediating measures regarding self-efficacy and attitude to parenthood, but not in the primary outcome measure quality in relation to the child as this is not applicable. The group leader checks the inclusion criteria for potential participants before the invitation to the group is carried out.

What selection criteria will be used for exclusion?

Parents who do not currently have or have previously had contact with their children are not included in the measure of the relationship with the child/child. The detained parent who has not or has not had contact with his/her children can be included in measures regarding self-efficacy and attitude to parenthood Has committed crimes against the child or violent crimes against the other parent Lack sufficient knowledge of the Swedish language in such a way that they can participate in the group, according to the group leader's assessment The group leader checks the exclusion criteria for potential participants before the invitation to the group is carried out.

Indicate the relationship between researchers and the research subjects

The researchers have no relation to the research subjects.

What insurance cover is available for the research subjects participating in the research project?

The participating parents in the parent groups are detained within the SPPS and they, as well as participating staff, are insured through the principal SPPS.

Describe the preparedness that exists to handle unexpected side findings or events during the research process that can jeopardize the safety of research subjects

In cases where group participants experience discomfort associated with group meetings or completing questionnaires, group leaders are on hand for conversation and support. Group leaders are in contact with group leader trainers for discussions and support regarding any situations that arise in connection with group meetings and group leaders are in contact with researchers for discussions about possible situations that arise in connection with data collection.

Will financial compensation or other benefits be paid to the research subjects?

Yes

What financial compensation will be paid and when?

Compensation of SEK 200 will be applied to the detained parents who participate in the interviews conducted after the intervention (approximately 15 parents from the intervention group). The compensation is deposited on each parent's kiosk card at the institution.

Information and consent

Will the research subjects be informed about the research project and asked if they want to join or not?

Yes

How, when (at what stage) and by whom are the research subjects informed and approached with a question to participate?

participating prison managers, group leaders and parents in custody will be informed and consent to participation or not. Institutional managers are informed when recruiting institutions June-September 2019 via e-mail, written information sheets, oral presentation of the study and by telephone call. The head of the institution decides whether the institution should participate and only after such a decision can information and the opportunity to consent be offered to clients deprived of liberty. Group leaders are informed when recruiting institutions June-September 2019 via email, written information sheets, oral presentation of the study and by telephone call. Group leaders and managers are informed by the researchers and have the opportunity to contact researchers for questions throughout the study period via email or phone. Parents in detention are informed through written information material, orally by group leaders after the institution has been recruited (August - September 2019). Parents have the opportunity to ask questions to the group leader continuously during the recruitment period. The group leaders are in contact with the researchers and can always convey questions from parents of researchers to get detailed and accurate answers. The participating parents in the group who are not included in the evaluation (due to not meeting the inclusion criteria or because they themselves do not agree) are informed of the audio recording of group meetings (via the same information sheet attached to the consent to participation in the evaluation) and specifically consent to the audio recording of the meetings. Unless all participants in the group agree to audio recording, audio recording is not carried out.

Will children under the age of 18 be included in the research project?

No

Will research subjects, whose opinion due to illness, mental disorder, weakened state of health or some other similar relationship cannot be obtained, be included in the research project?

No

Register data

Will the project request information from an existing register?

No

Biological material

Does the project plan to use biological material from one or more existing sample collections?

No

Will biological material be newly collected for the project?

No

Results from animal experiments

Are there relevant results from animal testing?

Non applicable

Reporting for results

How is access to data guaranteed for the research principal and participating researchers?

Responsible for project implementation has full access to data. Data will be saved on a server with limited availability at Karolinska Institutet. Paper questionnaires will be stored in locked, fireproof cabinets at Karolinska Institutet. Audio recordings of group meetings will be transferred from the Prison and Probation Service/group leader to the responsible researcher via a shared folder in a cloud solution secured by Karolinska Institutet (owncloud) where group leaders have access to the cloud through individual passwords and can only upload material.

Who is responsible for the data processing and presentation of the results in writing?

Data are processed and results are reported in writing by the responsible researchers for the project

How and when are the results planned to be published?

Results will be published in scientific journals during 2021-2022. Furthermore, the results will be presented at relevant scientific conferences during the same annual span. The findings will also be reported to the SPPS for which a report will be written. Furthermore, the findings will be presented to relevant professional groups within the Prison and Probation Service as well as parents who participated through seminars within the Prison and Probation Service and in writing.

In what way is the research subjects' right to privacy guaranteed when the material is published?

All prisons and participants will be assigned a code. All data will be anonymized and all presentation of results will take place at the group level where no individuals will be identifiable. Quantitative results are reported at the group level. In the qualitative data, quotes are indicated by participant code in which no quotes in which there may be an opportunity to derive to a specific person will be accounted for.

Economic conditions

Report any financial agreements with contributors or other funders (name and amount)

Funding has been received from the Solstickan Foundation for the period 2019-2020 with a total of SEK 850,000 and from the Sven Jerring Foundation for 2019 with a total of SEK 150,000. The remuneration covers all costs for personnel, materials, as well as compensation to participants.

Report on the research institution’s, principal investigator's and participating researchers' own financial interests

No financial interests.
